# An Incremental Clustering Algorithm with Pattern Drift Detection for IoT-Enabled Smart Grid System

**DOI:** 10.3390/s21196466

**Published:** 2021-09-28

**Authors:** Zigui Jiang, Rongheng Lin, Fangchun Yang

**Affiliations:** 1 School of Software Engineering, Sun Yat-Sen University, Zhuhai 519082, China; jiangzg3@mail.sysu.edu.cn; 2 State Key Laboratory of Networking and Switching Technology, Beijing University of Posts and Telecommunications, Beijing 100876, China; fcyang@bupt.edu.cn

**Keywords:** incremental learning, data stream clustering, load pattern, smart meter data

## Abstract

The IoT-enabled smart grid system provides smart meter data for electricity consumers to record their energy consumption behaviors, the typical features of which can be represented by the load patterns extracted from load data clustering. The changeability of consumption behaviors requires load pattern update for achieving accurate consumer segmentation and effective demand response. In order to save training time and reduce computation scale, we propose a novel incremental clustering algorithm with probability strategy, ICluster-PS, instead of overall load data clustering to update load patterns. ICluster-PS first conducts new load pattern extraction based on the existing load patterns and new data. Then, it intergrades new load patterns with the existing ones. Finally, it optimizes the intergraded load pattern sets by a further modification. Moreover, ICluster-PS can be performed continuously with new coming data due to parameter updating and generalization. Extensive experiments are implemented on real-world dataset containing diverse consumer types in various districts. The experimental results are evaluated by both clustering validity indices and accuracy measures, which indicate that ICluster-PS outperforms other related incremental clustering algorithm. Additionally, according to the further case studies on pattern evolution analysis, ICluster-PS is able to present any pattern drifts through its incremental clustering results.

## 1. Introduction

The smart grid system has been developing with the integration of massive new technologies, such as Internet of Things (IoT), Blockchain, and Artificial Intelligence (AI) [1,2,3]. Diverse IoT devices and frameworks are applied on smart grid to support data collection, transmission [4], real-time monitoring [5], etc. Blockchain technologies can provide decentralization, trust, and an incentive mechanism for improving the cybersecurity of smart grid system [6,7,8]. Compared with AI, the applications of AI methods including machine learning and deep learning are usually used to process and analyze data for decision-making, such as electric load forecasting [9,10], electric consumer categorization [11], and anomaly detection [12]. In such a smart grid system, the smart meter is an essential IoT device that records energy consumption data for further understanding, managing, planing, and optimizing power demands of electric consumers [13,14].

Smart meter data, also called electricity load data, are data streams that record the electricity consumption behaviors of consumers at regular intervals. They can be used for various studies and applications in smart grid, such as load forecasting, load profiling [15], anomaly detection [16,17], consumer categorization [18], and energy disaggregation [19]. In the studies of load profiling, one significant purpose is to extract the typical electricity consumption patterns, which is usually called load patterns, of every consumer based on load data clustering [20]. Most of works on load data clustering focus on the clustering problem of static load data. However, we notice that updating load patterns based on new load data is essential because electricity consumption behaviors may be changeable and inaccurate load patterns can cause wrong decisions. Although load patterns can be updated by conducting repetitive clustering on overall load data including the new ones, this leads to extra computation and storage, especially in batch-oriented data processing. In that case, incremental learning, which refers to learning from streaming data that arrive over time [21], can be a better solution as it can make full use of the historical information, reduce the training scale, and save training time [22]. Moreover, there are also some special clustering algorithms designed for data streams mining [23]. However, few of them are designed for high-dimensional smart meter data streams so that it is necessary to find out an effective incremental clustering algorithm to update load patterns, especially for end consumers with limited resources.

**Figure 1:**
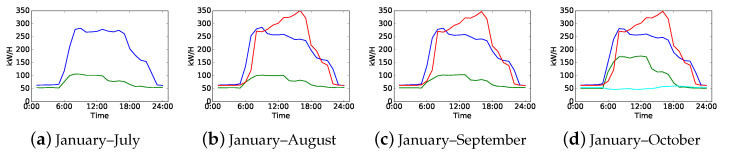
An example of electricity load patterns drift. (**a**) Load patterns of load data from January to July; (**b**–**d**) load patterns of load data that increase monthly comparing with the former one. All load patterns are obtained by daily load curve clustering.

In real-world industry and our daily lives, the electricity consumption behaviors of consumers may change over time. Some consumers keep their patterns for a long period while others may change frequently. An example of load pattern drift is shown in Figure 1. Each curve denotes a typical load pattern of the same electricity consumer, and the curves in the same color in different subfigures indicate the same load pattern. This consumer has two typical load patterns from January to July, which means that this consumer has a stable electricity consumption behavior. Then, it can be observed that the load patterns drift twice. The first drift happened in August shown by the red curve in Figure 1b, which indicates that this consumer has a new electricity consumption behavior. The second drift happened in August shown by the cyan curve in Figure 1d. Then, this consumer has four electricity consumption behaviors since October.

Once we extract the load patterns from the static electricity load data in a certain period, these load patterns are fixed unless they are updated. It is possible that there are some new load patterns that denote consumer behavior drift in the following periods, so that we should update the previously obtained load patterns by adding the new ones. However, consumer behaviors are complex. It is still uncertain that all coming load patterns are new, which means that some load patterns may exist in the previously obtained load patterns and others may not. In that case, we cannot simply add each load pattern extracted from the new coming data or assign them to any existing load patterns. How to update load patterns accurately is the main challenge of our incremental clustering problem.

Therefore, this work proposes an incremental clustering algorithm with probability strategy, which is named ICluster-PS. We assume that this algorithm can deal with smart meter data streams to update load patterns efficiently for every end-consumer through facilities with limited time and space. The incremental clustering algorithm of ICluster-PS includes three phases: load pattern extraction, load pattern intergradation, and load pattern modification. Load pattern extraction is a preparation to extract load patterns from new electricity load data, which are preprocessed as daily load curves. Load pattern intergradation and modification is an novel approach for determining whether or not we should create a new load pattern and optimize *K* for the number of updated load patterns. A short paper of this work is published in [24], and we revise and extend it by adding more details of the algorithm, experiments and pattern evolution analysis in this paper. The main contributions of this work are summarized as follows:

- We consider the problem of load pattern update based on smart meter data streams, and propose an incremental clustering algorithm for continuously updating load patterns. It is significantly helpful for learning electricity consumption behaviors in smart grid field.
- In the incremental clustering algorithm, we propose a probability strategy on distance measure for optimizing the performance of incremental clustering, and also consider updating parameter to conduct continuous incremental clustering with new coming data.
- We evaluate both accuracy and clustering validity of our algorithm on a real-world dataset, which contains 17,776 commercial and residential electricity consumers in various districts. The results indicate that ICluster-PS is closed to the performance of the non-incremental clustering based on overall daily load curves and outperforms other related incremental clustering algorithm in terms of both clustering validity and accuracy.
- The load pattern evolution can be clearly presented by the incremental clustering results, in which we are able to detect any pattern drifts or anomalies of electricity consumers.

The rest of this paper is organized as follows. Section 2 briefly reviews the related works. Section 3 provides the preliminary for Section 4, which introduces the details of the proposed incremental clustering algorithm. Experimental settings are presented in Section 5, and results with evaluation are discussed in Section 6. Finally, we conclude this work in Section 7.

## 2. Related Work

This section briefly reviews the most relevant related works in terms of load pattern extraction, incremental learning algorithms, and data stream clustering. Electricity consumer load pattern extraction is one of the most important research areas in smart grid, while incremental learning and data stream clustering are two related research areas in machine learning and data mining. However, there are few works that consider the problem how to conduct an incremental learning for electricity consumer load pattern extraction. Some relevant research works are compared in Table 1.

**Load Pattern Extraction.** Load pattern extraction is an unsupervised clustering problem. There are two types of clustering methods for load data clustering: direct clustering and indirect clustering [15]. In direct clustering, load data are directly used in clustering without any additional dimension reduction or data preprocessing methods. There are many classical clustering algorithms for load data clustering, such as *K*-means, fuzzy *K*-means, self-organizing map (SOM), and support vector clustering (SCV) [36,37,38]. As for indirect clustering, researchers usually pay more attentions to dimension reduction, feature extraction and feature construction methods for load data preprocessing. In [25], the authors constructed three new types of features. Their work indicates that the clustering performance of constructed features outperforms the one of default features. In [26], two variations of *K*-means algorithm with four proposed dimension reduction methods are applied to the clustering process in load profiling. A fused load curve clustering algorithm based on wavelet transform (FCCWT) is proposed in our previous work [39]. This algorithm first applies a multi-level wavelet transform to daily load curves for dimension reduction, and then fuse the *K*-means clustering results of both normalized approximation signals and detail signals, which are two outputs of wavelet transform, to gain an optimized clustering result.

**Incremental Learning Algorithms.** In recent years, incremental and online learning gain more attentions especially in big data and data stream areas [40,41]. There are many incremental learning algorithms based on ν-support vector regression, support vector machines (SVM), random forest (RF), neural networks, etc. [27,42,43,44]. An incremental support vector machine (ISVM) with Markov resampling (MR-ISVM) is introduced in [22] to study how dependent sampling methods influence the learning ability of ISVM. However, most of incremental learning algorithms study supervised classification without adding new classes. Although an incremental learning based on RF is studied to incrementally learn new classes for large-scale image [27], this method adds new classes into the trees without judging whether or not the coming classes are new. In [28], the authors proposed an incremental algorithm based on fast finding and searching of density peaks (CFS), named ICFKM, for clustering large data in industrial IoTs. Two challenges—how to integrate new clusters into the previous one and how to update the clustering centers—are solved in ICFKM, which seems to be useful for our incremental clustering problem. However, CFS has relatively strong subjectivity for selecting cluster centers based on the decision graph [45] so that it cannot applied in batch-oriented data processing. Moreover, CFS does not work well on relatively high-dimensional data. Many clusters may be missed by CPS because it only considers the global structure of data [46]. As time-series electricity load data have relatively high dimensions, ICFKM cannot be directly adopted for updating load patterns.

**Data Stream Clustering.** Clustering data streams requires the capability of partitioning observations continuously within limited memory and time [47]. Most data stream clustering algorithms consist of an online step that incrementally processes the data stream and produces summary statistics, and an offline step that summarizes data to generate clusters by traditional batch clustering algorithms [48]. There are various classic data stream clustering algorithms, such as Stream, CluStream, StreamKM++, DenStream, and HPstream. Both HPstream [29] and incPreDeCon [30] can deal with high-dimensional data streams. The former algorithm is based on *K*-means, while the later one is based on PreDecom which is a density-based clustering algorithm and requires too many parameters to be run efficiently. In [31], the authors introduced a data stream clustering based on Fuzzy *C*-mean algorithm and entropy theory. In [32], the authors developed algorithms for clustering high-dimensional dynamic data streams, whereas the algorithms are based on the assumption that no insertions of data that are already in the dataset, which may be not consistent with our load data. Meanwhile, the efficiency of these proposed algorithms is only evaluated by a 2D implementation. In [33], a fully online clustering algorithm is proposed for clustering evolving data streams into arbitrarily shaped clusters (CEDAS), which is also a density-based clustering algorithm. In [34], a density-based clustering algorithm called DStream-GC is designed for discovering gradual moving object clusters pattern from trajectory streams. In [35], a self-organizing incremental neural network (SOINN+) is developed for unsupervised learning clusters with arbitrary shapes from noisy data. Although some algorithms are incremental methods or declare that they can process high-dimensional data streams, their validity and efficiency on load pattern extraction and update require further evaluation. For example, density-based clustering algorithms may not achieve an excellent performance in the experiments of high-dimensional load curve clustering.

In summary, the works on load pattern extraction do not consider the incremental learning problem in their clustering algorithm, while the existing incremental learning or data stream clustering algorithms are not designed for load clustering. Therefore, it is essential to provide an incremental clustering algorithm for our load clustering problem.

## 3. Preliminary

Before introducing our method, we should first give the problem formulation and several important mathematical notations, shown in Table 2. We also briefly present the method used for load pattern extraction, which is the base of electricity consumer behavior learning.

### 3.1. Problem Formulation

For an electricity consumer, let $X_{0}=\{x_{01},x_{02},\ldots ,x_{0N_{0}}\}\;$$\in R^{d\times N_{0}}$ where $x_{0i}$ is *d*-dimensional vector be the electricity load data and $N_{0}$ be the number of days contained in the dataset $X_{0}$. We can extract the load patterns from these data by conducting daily load curve clustering.

**Definition** **1** (Daily Load Curve)**.** *A daily load curve $x_{0i}\;=<\;\;x_{0i1},x_{0i2},\;\ldots \;,x_{0id}\;\;>$ where $1\leq i\leq N_{0}$ is a d-dimensional vector that presents the electricity power consumption of one consumer in one day. It is recorded by a smart meter at a regular interval, which usually is 1 h, 30 min, or 15 min.*

**Definition** **2** (Load Pattern)**.** *Given a set of daily load curves $X_{0}=\left\{x_{01},x_{02},\ldots ,x_{0N_{0}}\right\}\in R^{d\times N_{0}}$, we apply a load curve clustering to $X_{0}$ and obtain a set of clusters $C_{0}\;=\;\left\{C_{01},C_{02},\ldots ,C_{0K_{0}}\;\right\}$. Let $A_{0}\;=\left\{\mu_{01},\mu_{02},\ldots ,\mu_{0K_{0}}\right\}\in R^{d\times K_{0}}$ be the set of cluster centers of $C_{0}$, and each $\mu_{0i}$ is called a load pattern that denotes one typical electricity power consumption behavior feature of the consumer. Every electricity consumer may have one or several load patterns.*

As $X_{0}$ contains $N_{0}$ daily load curves which are divided into $K_{0}$ clusters, let $n_{0i}$, $1\leq i\leq K_{0}$, be the number of daily load curves contained in the cluster $C_{0i}\in C_{0}$, and we obtain $\sum_{i=1}^{K_{0}}n_{0i}=N_{0}$. Then, we can give the definition of the probabilities of load patterns.

**Definition** **3** (Probability of Load Pattern)**.** *The probability of a load pattern $\mu_{0i}$ denotes the percentage of the daily load curves represented by $\mu_{0i}$ in the whole daily load curve dataset $X_{0}$. Let $P_{0}=\left\{p_{01},p_{02},\ldots ,p_{0K_{0}}\right\}$ be the set of probabilities of load patterns $A_{0}$, then*

(1) $$p_{0i}=\frac{n_{0i}}{N_{0}},$$

where $\sum_{i=1}^{K_{0}}p_{0i}=1$ and $1\leq i\leq K_{0}$.

After obtaining a set of load patterns $A_{0}$ based on $X_{0}$, a new set of daily load curves $X_{1}=\left\{x_{11},x_{12},\ldots ,x_{1N_{1}}\right\}\in R^{d\times N_{0}}$ comes due to the continuous electricity power consumption. We aim to obtain a set of updated load patterns $A_{1}=\left\{\mu_{11},\mu_{12},\ldots ,\mu_{1K_{1}}\right\}\in R^{d\times K_{1}}$ based on the existing load patterns $A_{0}$ and the new daily load curves $X_{1}$. This means that we conduct an incremental clustering with $X_{1}$ and $A_{0}$ rather than an overall clustering with $\left[X_{0},X_{1}\right]$.

As new sets of daily load curves continuously come, we can give a generalization of our incremental clustering problem. Let $A_{t-1}=\left\{\mu_{t-1,1},\mu_{t-1,2},\ldots ,\mu_{t-1,K_{t-1}}\right\}\in R^{d\times K_{t-1}}$ be the existing load patterns and $X_{t}=\left\{x_{t1},x_{t2},\ldots ,x_{tN_{t}}\right\}\in R^{d\times N_{t}}$ be the new set of daily load curves, we aim at proposing an incremental clustering algorithm that can obtain a set of updated load patterns $A_{t}\;=\;\left\{\mu_{t1},\mu_{t2},\ldots ,\mu_{tK_{t}}\;\right\}\in R^{d\times K_{t}}$, which equals or approximates to the load patterns extracted directly from overall daily load curves $X=\{X_{0},X_{1},\ldots ,X_{t}\}$.

### 3.2. Load Pattern Extraction

**Figure 2:**
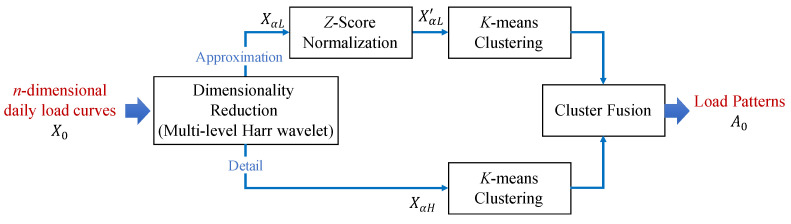
Diagram of non-incremental clustering algorithm FCCWT [39] for load pattern extraction.

Load pattern extraction is based on the clustering of daily load curves in this work. We adopt a fused load curve clustering algorithm called FCCWT [39] to extract the load patterns. The diagram of FCCWT is illustrated in Figure 2. This algorithm is designed specially for load clustering based on time-series electricity load data in our previous work. It conducts an indirect clustering, in which daily load curves are transformed into approximation signals and detail signals by a multi-level Harr wavelet before the load curve clustering for dimension reduction. Moreover, the approximation signals $X_{\alpha L}$ and detail signals $X_{\alpha H}$ are clustered separately and then fused to avoid information loss caused by the dimension reduction and improve the clustering performance. Although this algorithm is non-incremental, it provides a higher clustering validity comparing with other related methods.

## 4. Incremental Consumer Behavior Learning

In this section, we introduce the incremental clustering algorithm used for electricity consumption pattern learning. First, we present an overview of the incremental clustering algorithm. Second, we optimize this algorithm by a novel probability strategy in order to improve the incremental clustering performance. Third, several parameters are updated for the following continuous incremental clustering. Finally, we give the generalization of our optimized incremental clustering with the analysis of its asymptotic time complexity.

### 4.1. Incremental Clustering Algorithm

**Figure 3:**
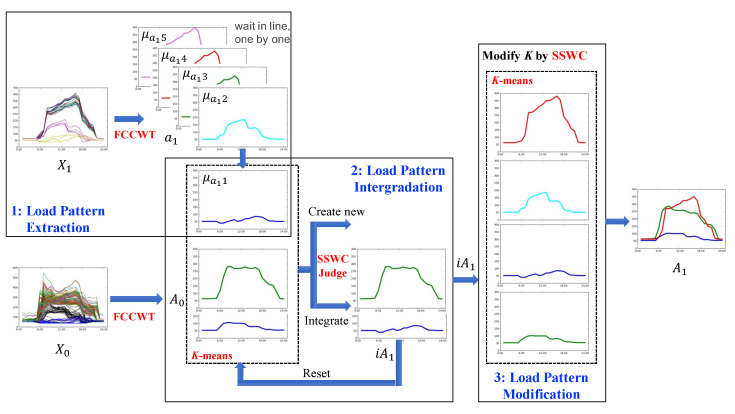
An illustration of the incremental clustering algorithm, including (1) load pattern extraction, (2) load pattern intergradation, and (3) load pattern modification. The inputs are the existing load patterns $A_{0}$ and new daily load curves $X_{1}$, and the output is the updated load patterns $A_{1}$.

As presented in Section 3, the inputs are the existing load patterns $A_{0}$ and new daily load curves $X_{1}$, while the output is the updated load patterns $A_{1}$. The main challenge of our problem is how to determine whether to create a new load pattern. As consumer behaviors are complex, it is uncertain that there are any different load patterns in $X_{1}$ comparing with $A_{0}$. We cannot conduct a simple clustering by regarding all $\mu_{0i}\in A_{0}$ as the cluster centers. As a result, a novel incremental clustering algorithm is proposed to intergrade the load patterns of $X_{1}$ into $A_{0}$. This model is able to determine whether integrating a load pattern into a $\mu_{0i}$ or keeping it as a new load pattern. An illustration of the incremental clustering algorithm is presented in Figure 3, which contains three phases: load pattern extraction, load pattern intergradation, and load pattern modification. As the example shown in Figure 3, the set of existing load patterns $A_{0}$, which is extracted from $X_{0}$, contains two load patterns. Then, we extract five new load patterns from new daily load curves $X_{1}$ and intergrade them with the two existing load patterns one by one. For the intergration of $\mu_{a_{1},1}$, we obtain an existing load pattern and an intergrated load pattern. After five times of load pattern intergradation and one extra load pattern modification, we finally obtain four updated load patterns.

Given a set of existing load patterns $A_{0}=\left\{\mu_{01},\mu_{02},\ldots ,\mu_{0K_{0}}\right\}$ and a set of new daily load curves $X_{1}=\left\{x_{11},x_{12},\ldots ,x_{1N_{1}}\right\}$, we describe these phases in detail.

**Load pattern extraction.** We need to process the new daily load curves $X_{1}=\{x_{11},x_{12},$$\ldots ,x_{1N_{1}}\}$ before the load pattern intergradation. A fused load curve clustering algorithm FCCWT [39], which is our previous work designed specially for daily load curve clustering, is applied to $X_{1}$. Then, we obtain the set of its load patterns $a_{1}=\left\{\mu_{a_{1}1},\mu_{a_{1}2},\ldots ,\mu_{a_{1}K_{a_{1}}}\right\}$. The corresponding probability of $a_{1}$ is $P_{a_{1}}=\left\{p_{a_{1}1},p_{a_{1}2},\ldots ,p_{a_{1}K_{a_{1}}}\right\}$, where $p_{a_{1}i}=n_{a_{1}i}/N_{1}$ and $1\leq i\leq K_{a1}$.

**Load pattern intergradation.** Let $iA_{1}$ denote the result of load pattern intergradation, and $iA_{1}$ is initialized as $iA_{1}=A_{0}$. We combine the *i*th load pattern $\mu_{a_{1}i}\in a_{1}$ with all load patterns in $iA_{1}$, which is denoted as $\left[iA_{1},\mu_{a_{1}i}\right]$. Then, two *K*-means clusterings are performed on $\left[iA_{1},\mu_{a_{1}i}\right]$ with $K=K_{0}$ and $K=K_{0}+1$, respectively. We evaluate their clustering results by the Simplified Silhouette Width Criterion (SSWC), which is one variant of Silhouette Width Criterion (SWC) index [39,49]. Then, the SSWC values of the clustering results when $K=K_{0}$ and $K=K_{0}+1$ are denotes as ${\mathrm{SSWC}}_{K=K_{0}}$ and ${\mathrm{SSWC}}_{K=K_{0}+1}$, respectively.

Given $\left[iA_{1},\mu_{a_{1}i}\right]=\left\{\mu_{1},\mu_{2},\ldots ,\mu_{K_{0}},\mu_{K_{0}+1}\right\}$ and its clustering result $C=\{c_{1},c_{2},\ldots ,c_{K}\}$ with the set of corresponding cluster centers $\bar{A}=\left\{{\bar{\mu }}_{1},{\bar{\mu }}_{2},\ldots ,{\bar{\mu }}_{K}\right\}$, the SSWC is calculated as the average of the Simplified Silhouette of the individual load pattern $\mu_{j}$ over $j=1,2,\ldots ,K_{0}+1$.

(2) $${\mathrm{SSWC}}_{K}=\frac{1}{K_{0}+1}\sum_{j=1}^{K_{0}+1}S_{\mu_{j}}=\frac{1}{K_{0}+1}\sum_{j=1}^{K_{0}+1}\frac{b_{c_{r},\mu_{j}}-a_{c_{r},\mu_{j}}}{\max\{a_{c_{r},\mu_{j}},b_{c_{r},\mu_{j}}\}},$$

where $a_{c_{r},\mu_{j}}$ is the distance between $\mu_{j}$ and the center of cluster $c_{r}\in C$, while $b_{c_{r},\mu_{j}}$ is the closest distance between $\mu_{j}$ and the centers of other clusters in *C* except for $c_{r}$. They are calculated as follows:

(3) $$a_{c_{r},\mu_{j}}=\mathrm{dist}\left(\mu_{j},{\bar{\mu }}_{r}\right),$$

(4) $$b_{c_{r},\mu_{j}}=\min\left\{\mathrm{dist}\left(\mu_{j},{\bar{\mu }}_{w}\right)\right\},$$

where 1≤r,w≤K and r≠w. *K* refers to the parameter of clustering conducted on $\left[iA_{1},\mu_{a_{1}i}\right]$. Then, we obtain ${\mathrm{SSWC}}_{K=K_{0}}$ and ${\mathrm{SSWC}}_{K=K_{0}+1}$ according to Equations (2)–(4). There are two situations when comparing ${\mathrm{SSWC}}_{K=K_{0}}$ and ${\mathrm{SSWC}}_{K=K_{0}+1}$.

(1) ${\mathrm{SSWC}}_{K=K_{0}}\geq {\mathrm{SSWC}}_{K=K_{0}+1}$ implies that the clustering performance of $K=K_{0}$ is equal or superior to the performance of $K=K_{0}+1$. As a result, we do not keep the *i*th load pattern $\mu_{a_{1}i}$ as a new load pattern, and adopt the set of cluster centers when $K=K_{0}$ as the integrating result of $\left[iA_{1},\mu_{a_{1}i}\right]$.

(2) ${\mathrm{SSWC}}_{K=K_{0}}<{\mathrm{SSWC}}_{K=K_{0}+1}$ implies that $K=K_{0}+1$ results in a better clustering performance than $K=K_{0}$ does. In that case, we keep the *i*th load pattern $\mu_{a_{1}i}$ as a new load pattern, and adopt the set of cluster centers when $K=K_{0}+1$ as the integrating result of $\left[iA_{1},\mu_{a_{1}i}\right]$.

After the above comparison and judgment, the set $iA_{1}$ is reset with the integrating result of $\left[iA_{1},\mu_{a_{1}i}\right]$. Each $\mu_{a_{1}i}$ over $i=1,2,\ldots ,K_{a_{1}}$ with iA is integrated gradually according to this procedure. Finally, we obtain the intergraded set $iA_{1}\;=\left\{i\mu_{11},i\mu_{12},\ldots ,i\mu_{iK_{iA_{1}}}\right\}$.

**Load pattern modification.** We perform a further modification on the intergraded set $iA_{1}$ to obtain an optimal incremental clustering result. As the the number of load patterns generally is within the range K∈[2,10] [25,39], multiple *K*-means clusterings are applied to $iA_{1}$ with *K* in the range of 2 to $\min\{K_{iA_{1}},10\}$, where $K_{iA_{1}}$ denotes the number of load patterns in $iA_{1}$. The SSWCs of $\min\{K_{iA_{1}},10\}-1$ times clusterings are calculated and compared with each other. Then we select the *K* with the largest SSWC as the optimal parameter, and regard the set of cluster centers with the selected optimal *K* as our target set of updated load patterns $A_{1}$.

We outline the incremental clustering of $A_{0}$ and $X_{1}$ in Algorithm 1, including the three phase mentioned above. In Algorithm 1, Line 1 is for load pattern extraction, Lines 2–11 conduct load pattern intergradation, and Lines 12–16 are for load pattern modification.
**Algorithm 1:** The incremental clustering algorithm
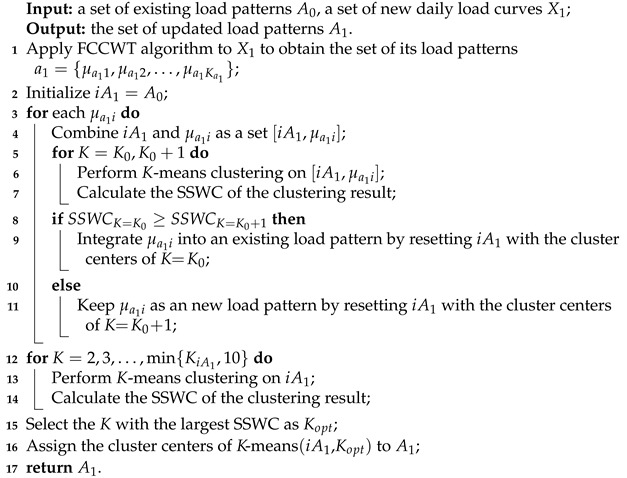



### 4.2. Optimization via Probability Strategy

Assume $A_{1}^{\prime }=\left\{\mu_{11}^{\prime },\mu_{12}^{\prime },\ldots ,\mu_{1K_{1}}^{\prime }\right\}\in R^{d\times K_{1}^{\prime }}$ is the load patterns extracted directly from the combined set $\left[X_{0},X_{1}\right]$, then $A_{1}^{\prime }$ is based on the non-incremental clustering of $N_{0}+N_{1}$ daily load curves. On the other hand, the incremental clustering algorithm shown in Algorithm 1 is based on the fusion of load patterns from both $A_{0}$ and $a_{1}$, which refer to only $K_{0}+K_{a_{1}}$ load patterns. Our purpose is to obtain an $A_{1}$ that equals or approximates to $A_{1}^{\prime }$. However, the simply *K*-means clustering algorithm with Euclidean distance is not appropriate to achieve this purpose.

It should be considered that the load patterns usually have different probabilities so that we should not treat them equally in the incremental clustering. Thus, an optimized distance measure with probability strategy is proposed for Algorithm 1, in which Euclidean distance measure is replaced with the proposed measure when performing both *K*-means clustering and SSWC calculation shown in Equation (3). It is assumed that this probability strategy can optimize Algorithm 1 to achieve an ideal $A_{1}$. Given a set of load patterns $A=\{\mu_{1},\mu_{2},\ldots ,\mu_{K}\}$ with the set of corresponding probability $P=\{p_{1},p_{2},\ldots ,p_{K}\}$, where $p_{i}=n_{i}/N$ and *N* is the number of daily load curves that *A* refers to, the optimized distance with probability strategy between $\mu_{i}$ and $\mu_{j}$ is calculated as follows:

(5) $$\begin{array}{c} {\mathrm{dist}}_{p}\left(\mu_{i},\mu_{j}\right)=p_{i}Np_{j}N||\mu_{i}-\mu_{j}{\left|\right|}_{2}=n_{i}n_{j}\left|\right|\mu_{i}-\mu_{j}{\left|\right|}_{2}, \end{array}$$

where $n_{i}$ and $n_{j}$ denote the numbers of daily load curves that $\mu_{i}$ and $\mu_{j}$ represent, respectively.

The cluster center ${\bar{\mu }}_{r}$ in *K*-means clustering with Euclidean distance is calculated as the mean of the objects that contained in the cluster:

(6) $${\bar{\mu }}_{r}=\frac{1}{m_{r}}\sum_{\mu_{\in }C_{r}}\mu_{r},$$

where $m_{r}$ is the number of μ contained in the cluster $C_{r}$. We set the probability of ${\bar{\mu }}_{r}$ with $p_{{\bar{\mu }}_{r}}=1/N$ when performing *K*-means clustering with the optimized distance. As a result, the optimized distance with probability strategy between $\mu_{i}$ and ${\bar{\mu }}_{r}$ is calculated as follows:

(7) $$\begin{array}{c} {\mathrm{dist}}_{p}\left(\mu_{i},{\bar{\mu }}_{r}\right)=p_{i}Np_{{\bar{\mu }}_{r}}N||\mu_{i}-{\bar{\mu }}_{r}{\left|\right|}_{2}=n_{i}\left|\right|\mu_{i}-{\bar{\mu }}_{r}{\left|\right|}_{2}. \end{array}$$

Similarly, the calculation of cluster center shown in Equation (6) should be rewritten as

(8) $${\bar{\mu }}_{r}=\frac{1}{\sum_{\mu_{\in }C_{r}}p_{r}N}\sum_{\mu_{\in }C_{r}}p_{r}N\mu_{r}=\frac{1}{\sum_{\mu_{\in }C_{r}}n_{r}}\sum_{\mu_{\in }C_{r}}n_{r}\mu_{r},$$

where $n_{r}$ denotes the number of daily load curves that the load pattern $\mu_{r}$ refers to, and $\sum_{\mu_{\in }C_{r}}n_{r}$ denotes the total number of daily load curves that all $\mu \in C_{r}$ refer to.

### 4.3. Updating Parameters

As new daily load data continuously grow with the electricity power consumption of consumers, we should update several essential parameters after one incremental clustering for the preparation of the next incremental clustering. The sets $X_{0}$ and $X_{1}$ contain $N_{0}$ and $N_{1}$ daily load curves, respectively. Their combined set $\left[X_{0},X_{1}\right]$ contains $N_{0}+N_{1}$ daily load curves totally. The incremental clustering on $\left[X_{0},X_{1}\right]$ gives the set of updated load patterns $A_{1}$ and the set of its corresponding clustering result $C_{1}=\left\{C_{11},C_{12},\ldots ,C_{1K_{1}}\right\}$. Let $P_{1}=\left\{p_{11},p_{12},\ldots ,p_{1K_{1}}\right\}$ be the set of corresponding probabilities of $A_{1}$, the probability of $\mu_{1r}\in A_{1}$ for the *r*th cluster $C_{1r}$ is updated as

(9) $$p_{1r}=\frac{n_{1r}}{N_{0}+N_{1}},$$

where $n_{1r}$ is the number of daily load curves that the load pattern $\mu_{1r}$ represents. We update $n_{1r}$ as follows:

(10) $$n_{1r}=\sum_{\mu_{0}\in C_{1r}}n_{0r}+\sum_{\mu_{a_{1}}\in C_{1r}}n_{a_{1}r},$$

where $\sum_{\mu_{0}\in C_{1r}}\;n_{0r}$ denotes the total number of daily load curves that all $\mu_{0i}\in A_{0}$ belonging to $C_{1}r$ represent, and $\sum_{\mu_{a_{1}}\in C_{1r}}\;n_{a_{1}r}$ denotes the one that all $\mu_{a_{1}i}\in a_{1}$ belonging to $C_{1}r$ represent. After the updating of $P_{1}$, $A_{1}$ is ready to be conducted in another incremental clustering with the next coming data set $X_{2}$.

### 4.4. Generalization of Incremental Clustering

In practice, there are continuous coming new daily load data sets $X_{1},X_{2},\ldots ,X_{t}$. The generalization of incremental clustering algorithm, which is based on the existing load patterns $A_{0}$ and new daily load curves $X=\{X_{1},X_{2}\ldots ,X_{t}\}$, is outlined in Algorithm 2. For $n_{sr}$ and $p_{sr}$ in the generalized algorithm, the updating equations shown in Equation (9) and Equation (10) become

(11) $$p_{sr}=\frac{n_{sr}}{\sum_{i=0}^{s}N_{i}},$$

(12) $$n_{sr}=\sum_{\mu_{s-1}\in C_{sr}}n_{s-1,r}+\sum_{\mu_{a_{s}}\in C_{sr}}n_{a_{s}r},$$

where $N_{i}$ is the number of daily load curves that $X_{i}$ contains, and $\mu_{s-1}$ and $\mu_{a_{s}}$ are the load patterns that belong to $A_{s-1}$ and $a_{s}$, respectively.

In Algorithm 2, the incremental clustering is continuously performed with the coming of $X_{s}$. This means that it is performed immediately once $X_{s}$ comes without waiting all $X_{s+1},X_{s+2},\ldots ,X_{t}$ come. Therefore, we can obtain the updated load patterns $A_{s}$ in time, and then the algorithm is paused until $X_{s+1}$ comes.
**Algorithm 2:** The generalization of Algorithm 1
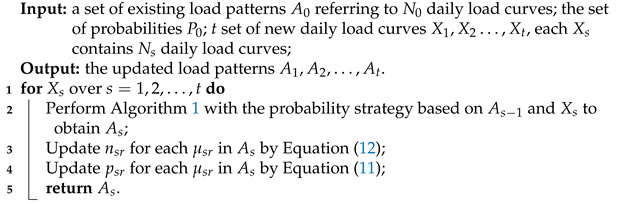



### 4.5. Complexity Analysis

The time complexity of FCCWT is O(NKT) where *N* is the number of daily load curves, *K* is the number of clusters and *T* is the number of iterations needed until convergence [39]. The time complexity of *K*-means is O(NdKT) while the one of SSWC calculation is O(NdK), where *d* is the size of dimensions of daily load curves. As the default of maximum *T* is usually set as 100, 200, or 300, we assume that all *T*s in Algorithm 1 are the same so that the time complexity can be analyzed more easily. Moreover, we also assume that all *K*s adopt the maximum value 10 due to K∈[2,10].

Based on the above assumptions, the asymptotic time complexities of load pattern extraction, load pattern intergradation and load pattern modification in Algorithm 1 are $O(KN_{1}T)$, $O(\left(2K^{3}+3K^{2}+K\right)d\left(T+1\right))$ and $O(Kd\left(T+1\right)\sum_{k=2}^{K}k)$, respectively. Therefore, the asymptotic time complexity of Algorithm 1 is

(13) $$\begin{array}{cc} \; & O\left(KN_{1}T+\left(2K^{3}+3K^{2}+K\right)d\left(T+1\right)+Kd\left(T+1\right)\sum_{k=2}^{K}k\right)\\ \; & =O\left(KN_{1}T+\left(2K^{3}+3K^{2}+K\sum_{k=1}^{K}k\right)d\left(T+1\right)\right). \end{array}$$

The time complexity of updating parameters is O(K) so that the asymptotic time complexity of Algorithm 2 is

(14) $$O\left(\left(KT\sum_{s=1}^{t}N_{s}+\left(2K^{3}+3K^{2}+K\sum_{k=1}^{K}k\right)td\left(T+1\right)+Kt\right)\right),$$

where $O(KT\sum_{s=1}^{t}N_{s})$ is the time complexity of *t* times FCCWT performed on $X_{s}$, $O(\left(2K^{3}\;+\;3K^{2}\;+\;K\sum_{k=1}^{K}k\right)td\left(T\;+\;1\right))$ is the time complexity of *t* times load pattern intergradation and modification, and O(Kt) is the time complexity of *t* times parameter updating.

As for non-incremental clustering, the time complexity of *t* times FCCWT on $\left[X_{0},X_{1},\cdot ,X_{s}\right]$ over s=1,2,…,t is

(15) $$\begin{array}{cc} \; & O\left((N_{0}+\sum_{s=0}^{1}N_{s}+\sum_{s=0}^{2}N_{s}+\ldots +\sum_{s=0}^{t}N_{s})KT\right)\\ \; & =O\left(\left(\left(t+1\right)N_{0}+tN_{1}+\left(t-1\right)N_{2}+\ldots +N_{t}\right)KT\right), \end{array}$$

which is sensitive to the size of *t*. Similarly, the time complexity of *t* times non-incremental clustering algorithm *K*-means on the same data is $O(\left(\left(t+1\right)N_{0}+tN_{1}+\left(t-1\right)N_{2}+\ldots +N_{t}\right)dKT)$. Comparing Equation (15) with the time complexities of two non-incremental clustering algorithms, it is suggested that the incremental clustering saves time and reduces the clustering scale when *t* is relatively large.

## 5. Experimental Settings

This section presents the experimental settings including datasets, evaluation criterion, and comparison methods in details. In evaluation criterion, an weighted mean error measure is proposed to evaluate the accuracy of the load patterns extracted by incremental clustering.

### 5.1. Datasets

The dataset used in the experiment refers to 14,976 commercial and 2800 residential electricity consumers in 936 counties of United States (Available online: https://openei.org/datasets/files/961/pub/ (accessed on 24 June 2019). Eight of 2808 residential consumers have missing data so that only the data of 2800 residential electricity consumers are used in the experiment). It contains 24-value daily load data over one year and records the electricity power consumption at every 1 h from 1:00 to 24:00 per day. As the proposed algorithm is designed for learning the electricity consumption patterns of a single consumer, the data of one electricity consumer can be regarded as a sub-dataset that leads to a sub-experiment. As a result, we conduct 17,776 sub-experiments totally. Moreover, three situations are considered for every sub experiment. We select 3 months, 6 months, and 9 months daily load data as the initial set $X_{0}$, respectively. The remaining data are divided by month and then regarded as $X_{1},X_{2},\cdots ,X_{t}$. For example, in the case of t=3, daily load data from January to September are selected as $X_{0}$, and the data of October, November, and December are regarded as $X_{1}$, $X_{2}$, and $X_{3}$, respectively.

### 5.2. Evaluation Criterion

We employ two types of measures including clustering validity indices and accuracy measures as the evaluation criterion in the experiment. Moreover, we propose an weighted mean minimum error measure for the accuracy measures.

**Clustering validity indices.** The clustering performance of the proposed method is also evaluated by diverse clustering validity indices including Davies–Bouldin index (DB), Dunn validity index (DVI), and SWC.

Let $C_{s}=\left\{C_{s1},C_{s2},\cdots ,C_{sK_{s}}\right\}$ be the corresponding clustering results of $A_{s}$, the clustering validity indices of $C_{s}$ follow the equations below:

(16) $$\mathrm{DB}\left(C_{s}\right)=\frac{1}{K_{s}}\sum_{r=1}^{K_{s}}\max_{w\neq r}\left\{\frac{{\bar{C}}_{sr}+{\bar{C}}_{sw}}{\left|\right|\mu_{sr}-\mu_{sw}\left|\right|}\right\},$$

(17) $$\mathrm{DVI}\left(C_{s}\right)\;=\;\frac{\min_{0<r\neq w<K_{s}}\;\;\left\{\min_{\forall x_{i}\in C_{sr},\forall x_{j}\in C_{sw}}\left\{\left|\right|x_{i}-x_{j}{\left|\right|}_{2}\right\}\right\}}{\max_{0<r\leq K_{s}}\max_{\forall x_{i},x_{j}\in C_{sr}}\left\{\left|\right|x_{i}-x_{j}{\left|\right|}_{2}\right\}},$$

(18) $$\mathrm{SWC}=\frac{1}{N}\sum_{j=1}^{N}\frac{b_{C_{sr},x_{j}}-a_{C_{sr},x_{j}}}{\max\{a_{C_{sr},x_{j}},b_{C_{sr},x_{j}}\}},$$

where ${\bar{C}}_{sr}$ and ${\bar{C}}_{sw}$ is the average within-group distance for $C_{sr}$ and $C_{sw}$, respectively; $x_{i}$ and $x_{j}$ denote two daily load curves contained in $\left[X_{0},X_{1},\ldots ,X_{s}\right]$, respectively; $N=\sum_{i=0}^{s}N_{i}$, $a_{C_{sr},x_{j}}$ denotes the mean distance of $x_{j}$ to all other daily load curves in $C_{sr}$; and $b_{C_{sr},x_{j}}$ denotes the minimum mean distance of $x_{j}$ to all daily load curves in $C_{sw}$, w≠r.

**Accuracy measures.** As we aim to obtain $A_{s}=\left\{\mu_{s1},\mu_{s2},\cdots ,\mu_{sK_{s}}\right\}$ that equals or approximates to $A_{s}^{\prime }=\left\{\mu_{s1}^{\prime },\mu_{s2}^{\prime },\cdots ,\mu_{sK_{s}^{\prime }}^{\prime }\right\}$, which is the load patterns extracted directly from $\left[X_{0},X_{1},\cdots ,X_{s}\right]$, we employ the accuracy measures for time-series forecasting to evaluate the load patterns in $A_{s}$ comparing with those in $A_{s}^{\prime }$. Both scale-dependent and percentage-based measures are employed, including Normalized Root Mean Square Error (NRMSE), Mean Absolute Error (MAE), and Symmetric Mean Absolute Percentage Error (sMAPE) [50,51]. However, both $A_{s}$ and $A_{s}^{\prime }$ contain several load patterns so that we propose a weighted mean minimum error based on the numbers of load patterns in $A_{s}$ and $A_{s}^{\prime }$.

(19) $$\mathrm{NRMSE}\left(\mu_{si}^{\prime },\mu_{sj}\right)=\sqrt{\frac{1}{d}\sum_{l=1}^{d}{\left(\frac{\mu_{si,l}^{\prime }-\mu_{sj,l}}{\mu_{si,l}^{\prime }}\right)}^{2}},$$

(20) $$\mathrm{MAE}\left(\mu_{si}^{\prime },\mu_{sj}\right)=\frac{1}{d}\sum_{l=1}^{d}|\mu_{si,l}^{\prime }-\mu_{sj,l}|,\;\;\;\;\;$$

(21) $$\mathrm{sMAPE}\left(\mu_{si}^{\prime },\mu_{sj}\right)=\frac{1}{d}\sum_{l=1}^{d}\frac{2\cdot |\mu_{si,l}^{\prime }-\mu_{sj,l}|}{\left(\right|\mu_{si,l}^{\prime }\left|+\right|\mu_{sj,l}\left|\right)}.\;\;$$

(1) $K_{s}^{\prime }\leq K_{s}$ indicates that the incremental clustering may cause extra load patterns. We calculate the minimum error for each $\mu_{sj}\in A_{s}$, which is the error between $\mu_{sj}$ and its most similar load pattern $\mu_{si}^{\prime }\in A_{s}^{\prime }$. Moreover, we weight the mean error by $K_{s}/K_{s}^{\prime }$ due to the extra load patterns.

(22) $$E\left(A_{s}^{\prime },A_{s}\right)=\frac{K_{s}}{K_{s}^{\prime }}\frac{1}{K_{s}^{\prime }}\sum_{j=1}^{K^{\prime }}\min_{\mu_{sj}\in A_{s}}\left\{\mathrm{meas}(\mu_{si}^{\prime },\mu_{sj})\right\},$$

where $\mathrm{meas}(\mu_{si}^{\prime },\mu_{sj})$ can be NRMSE, MAE, or sMAPE shown in Equation (19)–(21).

(2) $K_{s}^{\prime }>K_{s}$ indicates that the incremental clustering misses some load patterns. We calculate the minimum error for each $\mu_{si}^{\prime }\in A_{s}^{\prime }$, which is the error between $\mu_{si}^{\prime }$ and its most similar load pattern $\mu_{sj}\in A_{s}$. Similarly, we weight the mean error by $K_{s}^{\prime }/K_{s}$ due to the missing load patterns.

(23) $$E\left(A_{s}^{\prime },A_{s}\right)=\frac{K_{s}^{\prime }}{K_{s}}\frac{1}{K_{s}}\sum_{i=1}^{K}\min_{\mu_{si}^{\prime }\in A_{s}^{\prime }}\left\{\mathrm{meas}(\mu_{si}^{\prime },\mu_{sj})\right\}.$$

According to the definitions of these indices and measures, smaller Errors indicate higher accuracy and smaller DB indicates better clustering performance. On the contrary, the larger the DVI and SWC are the better the clustering performance is.

### 5.3. Comparison Methods

We adopt two algorithms FCCWT and *K*-means to conduct non-incremental clustering on $\left[X_{0},X_{1},\cdots ,X_{s}\right]$ over s=1,2,⋯,t, and then regard the load patterns with the optimal clustering performance as the baseline for evaluating the accuracy of other incremental clustering methods. Moreover, we also compare the clustering performance of the non-incremental clustering algorithms with our proposed method and other related incremental clustering methods. Methods compared in the experiments are summarized in Table 3.

## 6. Results and Evaluation

In this section, we first present and discuss the general incremental clustering performance and accuracy of comparison methods on data of all consumers. Then, a commercial consumer is randomly selected as a case for electricity consumption behavior patterns analysis. We also compare the mean runtime of incremental and non-incremental clustering algorithms to support the time complexity analysis in the former section. Furthermore, we conduct pattern evolution analysis based on the incremental clustering results of another randomly selected residential consumer.

### 6.1. Incremental Clustering Performance

We conduct the experiments of Algorithm 2 with t=3, t=6, and t=9, which means that three, six, and nine incremental clustering processes shown in Algorithm 1 are performed in one experiment, respectively. Both clustering performance and accuracy of the methods are compared for the incremental clustering performance. Although there are various types of consumers in the dataset, we still use the mean performance of all consumers to evaluate the comparison methods because most of the evaluation criteria are percentage-based.

Table 4 shows the mean clustering performance comparison of the methods on the data of 17,776 electricity consumers. The former two methods are non-incremental clustering methods while the later five methods are incremental. We first compare the mean clustering performance of incremental methods. According to the definitions of three clustering validity indices shown in Equation (16)–(18), the larger the DVI and SWC are the better the clustering performance is, while a smaller DB indicates better clustering performance. The optimal results of incremental methods are displayed in bold. The proposed method ICluster-PS shows the smallest DB values and largest SWC values in Table 4. Although the DVI values of ICluster-PS are slightly lower than the ones of HPStream when t=3 and t=6, the average clustering performance of ICluster-PS is optimal in all compared incremental clustering methods. Therefore, these results indicate that the proposed method ICluster-PS outperforms other incremental methods. The largest improvement of clustering performance comparing with other incremental clustering methods is 44.2%. On the other hand, ICluster-PS still requires improvement due to its lower clustering performance compared with the non-incremental clustering FCCWT and *K*-means that conduct clustering directly on overall daily load curves.

As FCCWT presents the optimal clustering performance in Table 4, we decide to adopt the load patterns obtained from FCCWT as the baseline for accuracy measure. Then, we can calculate the mean errors of the five incremental methods based on Equation (22) and Equation (23) using three different accuracy measures. The results, which are shown in Table 5, indicate that ICluster-PS has the optimal performance as the minimum error denotes the highest accuracy. The improvement of accuracy is between 29.8% and 66.0% comparing with other incremental clustering methods.

According to the results shown in Table 4 and Table 5, the better clustering performance and smaller errors of methods with probability strategy compared with those without the strategy prove the optimization of our proposed probability strategy. Moreover, the incremental clustering algorithm of ICluster-PS, especially load pattern intergradation and modification, improves both clustering performance and the accuracy of *K*-means based on the comparisons between ICluster and *K*-means with or without probability strategy. As for the three groups of mean errors with different *t*, it is noticed that the mean errors increase with the rise of *t*, which means that the errors may increasingly rise over the continuous incremental clustering. However, the three groups of the mean clustering performance present an opposite tendency. Therefore, it can be only suggested that the load patterns updated by incremental clustering may tend to deviate from the load patterns obtained by FCCWT over time.

In summary, the proposed incremental clustering algorithm, ICluster-PS, can achieve an acceptable accuracy with mean error less than 10% and an improved clustering validity via its designed model and probability strategy. This result indicates that we can provide an efficient response when consumers require consumption analysis via smart meter or other facilities with limited resource. Although our experiments set the data of one month as $X_{s}$, it can be set optionally by consumers in practical application.

### 6.2. Case Analysis

**Figure 4:**
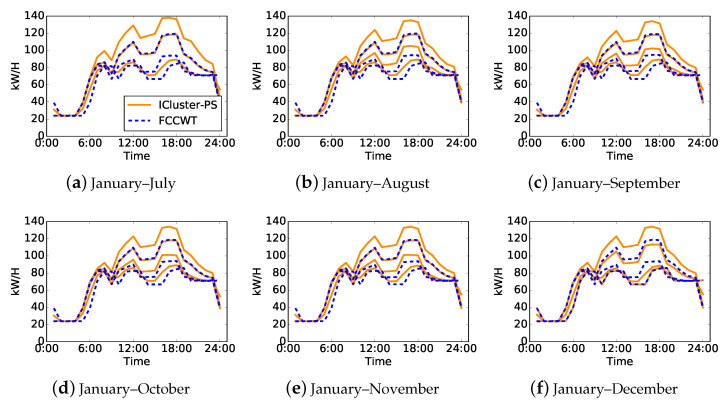
Load pattern comparison between ICluster-PS and FCCWT of one randomly selected electricity consumer when t=6. (**a**–**f**) Curves presenting the load patterns based on incremental or non-incremental clustering of $\left[X_{0},X_{1},\cdots ,X_{s}\right]$ over s=1,2,⋯,t.

A random electricity consumer is selected to be analyzed in detail for a further discussion of the proposed method and electricity consumer behaviors. The selected consumer is a full service restaurant, which have three typical load patterns based on the overall daily load curves. Figure 4 illustrates the load patterns obtained by ICluster-SP and FCCWT in the experiment when t=6. Each subfigure presents both the incremental and non-incremental cluster centers of the data $\left[X_{0},X_{1},\cdots ,X_{s}\right]$, where 1≤s≤t. The load patterns in solid line style denote the incremental cluster centers of ICluster-SP, while those in dashed line style denote the non-incremental cluster centers of FCCWT.

According to the clustering performance shown in Table 4, the load patterns of FCCWT are regarded as the accurate results. Note that these accurate load patterns are relatively stable and there is no distinct electricity consumption behavior drift happening to this consumer from July to December. The three typical load patterns of this consumer are distinct in terms of power degrees, starting time of the increase in the morning and ending time at night. The possible reasons for these distinctions are daylight saving time and seasonal influence. As for the incremental clustering results, their load patterns drift once on August shown in Figure 4b. Therefore, we can find out three typical load patterns in Figure 4a and four typical load patterns in other subfigures. These updated load patterns show similar patterns as the accurate ones if the power degrees of them are not taken into account. However, the distinct starting time of the increase in the morning shown by the accurate ones are not revealed by those of ICluster-PS until December, shown in Figure 4f.

**Figure 5:**
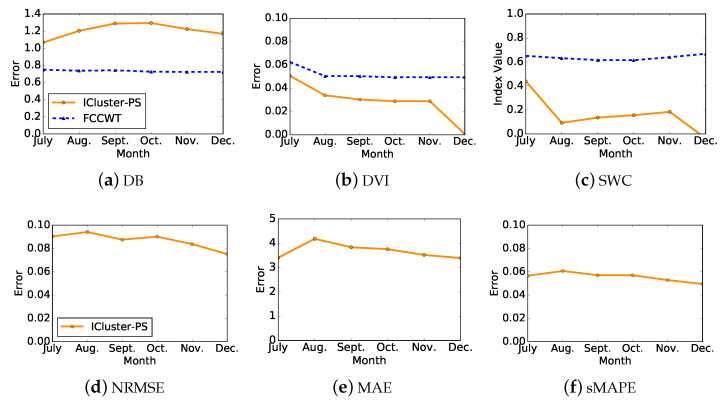
Errors and clustering validity indices comparisons between ICluster-PS and FCCWT of one randomly selected electricity consumer when t=6. Each curve contains six values referring to the evaluation of load patterns in Figure 4a–f, respectively.

In addition, we evaluate the load patterns by the same accuracy measures and clustering validity indices used in the former evaluation, the results of which are illustrated in Figure 5. Each curve contains six values which refer to the evaluation of load patterns in Figure 4a–f, respectively. Figure 5a–c presents the clustering performance of both ICluster-PS and FCCWT. FCCWT shows a relatively stable clustering performance while ICluster-PS shows slight fluctuation. The optimal clustering performance, especially for DVI and SWC, of ICluster-PS is presented in July. On the other hand, Figure 5d–f denotes the accuracy measures of ICluster-PS comparing with FCCWT so that there is only one curve in each subfigure. All three curves show an increase at first and then decrease after August. Different from the presentation of its clustering performance, their optimal accuracies are shown in December, which are in accord with the results shown in Figure 4.

Based on the observation of this case, ICluster-PS can achieve incremental clustering for load pattern updating, although it may provide an slightly unstable performance in terms of accuracy and clustering validity. This result is acceptable for providing efficient and effective updated electricity consumption patterns with time and space constraints.

### 6.3. Runtime Comparison

**Figure 6:**
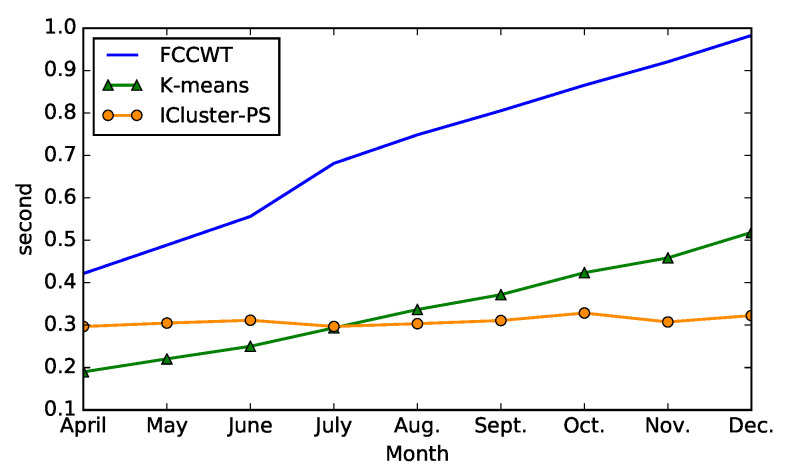
Mean runtime comparison of three methods on the data of 16 commercial consumers in 100 time experiments when t=9 (nine clusterings from April to December).

Apart from the time complexity analysis of both incremental and non-incremental clustering algorithms in Section 4, we also compare their runtime in the experiment to support this analysis. The algorithms, which are written in Python and run on 64-bit Windows 10 operating system with Intel Core i5-5300U CPU and 8 GB RAM, are performed on the data of 16 commercial consumers in a same randomly selected county. Figure 6 shows the mean runtime comparison of the methods when t=9. The comparison methods include the proposed incremental clustering algorithm ICluster-PS, and two non-incremental algorithms, FCCWT and *K*-means. Each algorithm is run 100 times in every clustering, which means that we run 16×9×100×3 times non-incremental or incremental clustering algorithms totally. According to Figure 6, it can be noticed that the runtime of ICluster-PS is stable and around 0.3 s while the the runtime of other two non-incremental clusterings increase with the rise of *t*. This result proves the time complexity analysis in Section 4, which is that the incremental clustering saves time when *t* is relatively large because it reduces the clustering scale. The runtime curve of ICluster-PS shows some slight fluctuations, which are caused by the small differences of the data in every month.

### 6.4. Pattern Evolution Analysis

**Figure 7:**
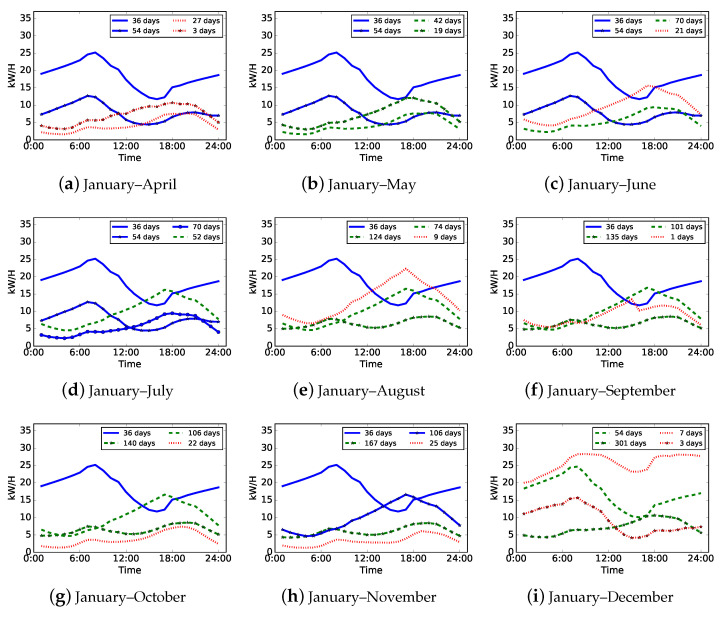
Updated load patterns of a residential consumer from April to December (t=9). Each subfigure shows the load patterns updated by one incremental clustering with one month adding new data based on the load patterns of previous months. Load patterns in blue and solid line style denote existed patterns, those in green and dashed line style denote updated patterns, and others in red and point line style denote new patterns.

We assume that our incremental clustering algorithm can be used to investigate the electricity consumption pattern evolution over time when conducting load pattern updates. As a result, we randomly select a residential consumer, who may have less stable consumption patterns than a commercial consumer, as a case for pattern evolution analysis. Figure 7 shows the updated load patterns of the selected residential consumer from April to December, which means that t=9 is set in the experiment of this case analysis. Each subfigure denotes the load patterns of one incremental clustering with one month adding new data based on the load patterns of previous months. For example, Figure 7a indicates the load patterns updated by the first incremental clustering based on the existed load patterns of January to March and new daily load data of April, Figure 7b indicates the load patterns updated by the second incremental clustering based on the load patterns shown in Figure 7a and new daily load data of May, etc. In Figure 7, we use curves with different colors, line styles, and markers to distinguish various types or meanings of updated load patterns. The curves in blue and solid line style denote the load patterns that exist in last month, which means that these load patterns are not affected by new adding data and do not drift in current month. The curves in green and dashed line style denote the load patterns that are updated by new adding data in current month and have drifts comparing with the ones in last month. The curves in red and point line style denote the load patterns which are completely new and only refer to the days in current month. Markers on curves are only used to label different load patterns.

**Figure 8:**
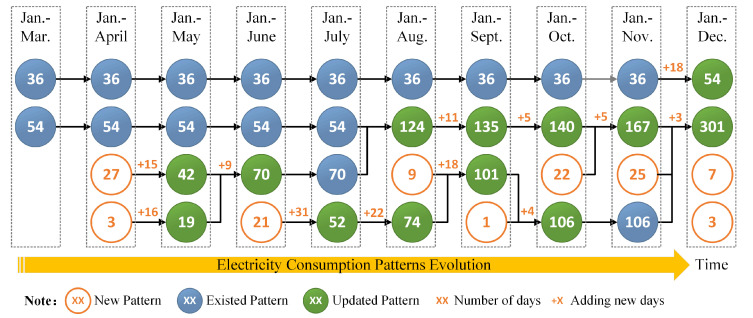
Electricity consumption patterns evolution of the case showed in Figure 7. The first column (left) denotes the load patterns extracted by non-incremental clustering with data from January to March. Other columns, each of which denotes the load patterns updated by incremental clustering based on one month adding new data and the load patterns shown in left column, are corresponding to Figure 7a–i, respectively.

Moreover, we draw another figure, Figure 8, to illustrate the pattern evolution of the case shown in Figure 7. In Figure 8, each circle with a number denotes a cluster or load pattern, and the number inside the circle denotes the number of days that the load pattern refers to. There are three types of circles, which represent existed load patterns, updated load patterns and new load patterns, respectively. The plus and number shown on an arrow denote the number of new days added to the load pattern after one incremental clustering. In fact, Figure 8 is in accordance with Figure 7. The first column in left of Figure 8 indicates two load patterns extracted by non-incremental clustering with load data from January to March. Other nine columns, each of which denotes four load patterns updated by an incremental clustering with adding new load data in current month and the load patterns in last month (shown in left column), are corresponding to Figure 7a–i, respectively.

The electricity consumption pattern evolution of the residential consumer is presented clearly according to Figure 7 and Figure 8. The residential consumer has two load patterns, which refer to 36 and 54 days, in the first three months of the year based on a non-incremental clustering with the data from January to March. Note that the load pattern with 36 days is unchanged until December. There are 18 new days in December that have similar shape with this load pattern so that they are added in this pattern and the number of days included in this pattern becomes 54. Then, it can be found that this load pattern drifts slightly based on the comparison of the curve with 36 days shown in Figure 7h and the curve with 54 days shown in Figure 7i. Another load pattern with 54 days at first is unchanged until August. Then, it is continually updated and merged with new days or other existed load patterns, and finally becomes a load pattern with 301 days (Jan–Dec), which is presented by the green dashed curve with star markers shown in Figure 7i. In total, nine new load patterns emerge in April, June, August, September, October, November, and December. Most of them are merged with other load patterns in next month. For instance, a new load pattern with nine days emerges in August, then it is merged with 18 new days in September and the other load pattern with 74 days (Jan–Aug), and finally becomes an updated load pattern that refers to 101 days (Jan–Sept). We note that some load patterns are merged after one or several incremental clustering. Why do different load patterns become one after one or a few months? The reason is that the increase in the number of data samples leads to the change of the optimal clustering results.

Based on the pattern evolution analysis and the further analysis on the dates of all days included in every load pattern, we can find out when and how this residential consumer drifts electricity consumption behaviors. In that case, this consumer can have a clear understand of her/his electricity demand and make an effective response to it. On the other hand, electricity suppliers or other agencies can also detect any anomalies once electricity consumers, especially commercial or industrial consumers, drift their consumption patterns significantly.

## 7. Conclusions and Future Work

This paper aims to achieve efficient demand response and consumer segmentation for both electricity end consumers and suppliers by incremental consumer behavior learning. It supposes that an effective incremental clustering algorithm would constantly updated load pattern data for electricity consumers with limited resource. Moreover, the incremental clustering algorithm should reduce the training scale and save time comparing with non-incremental clustering algorithms.

Therefore, we propose an incremental clustering algorithm with probability strategy, ICluster-PS, for updating load patterns based on smart meter data. We also provide parameter updating and algorithm generalization to ICluster-PS in order to continuously perform our algorithm with new coming data. The proposed algorithm is evaluated on real-world data. The experimental results prove the accuracy and validity of our incremental clustering algorithm, especially load pattern intergradation, modification, and probability strategy. It has less time complexity and runtime than non-incremental clustering algorithm. On the other hand, although ICluster-PS cannot provide load patterns that are the same as those extracted directly from the overall electricity load data, it achieves acceptable updated results when saving time, reducing the clustering scale and even making full use of the historical information. It also outperforms other related incremental algorithms or data stream clustering algorithms.

Moreover, we conduct additional case study of pattern evolution analysis by using our proposed algorithm. The analysis results indicate that our algorithm is able to detect load pattern drifts through its updated load patterns. In the future work, we plan to improve the performance of the incremental clustering algorithm and employ incremental consumer behavior learning for automatic and real-time load pattern evolution analysis and detection.

## Figures and Tables

**Table 1:** Comparison with existing research works.

Research Works	Year	Algorithms	Unsupervised	Data Types	Incremental	New Class
Xu et al. [22]	2018	SVM-based	No	Multiple	Yes	No
Jiang et al. [24]	2019	*K*-means-based	Yes	load data	No	-
Al-Otaibi et al. [25]	2016	feature construction	Yes	load data	No	-
Panapakidis et al. [26]	2015	*K*-means-based	Yes	load data	No	-
Marxer & Purwins [27]	2016	RF-based	No	image data	Yes	Yes
Zhang et al. [28]	2017	density-based	Yes	IoT data	Yes	Yes
Aggarwal et al. [29]	2004	*K*-means-based	Yes	data streams	-	-
Kriegel et al. [30]	2011	density-based	Yes	data streams	-	-
Zhang et al. [31]	2016	Fuzzy C-mean-based	Yes	data streams	-	-
Braverman et al. [32]	2017	*K*-median-based	Yes	data streams	-	No
Hyde et al. [33]	2017	density-based	Yes	data streams	Yes	-
Zhang et al. [34]	2019	density-based	Yes	trajectory streams	Yes	-
W. & Berrar [35]	2020	neural network-based	Yes	noisy data	-	-
**Proposed work**	2021	*K*-means-based	Yes	load data	Yes	Yes

**Table 2:** Several important mathematical notations.

Notations	Description
*X*	the set of overall daily load curves
$X_{s}$	the *j*th set of daily load curves, 0≤s≤t
$x_{si}$	the *i*th daily load curve in $X_{s}$, $1\leq i\leq N_{s}$
*d*	No. of dimensions of daily load curves
*t*	No. of coming new daily load curves
$N_{s}$	No. of daily load curves in $X_{s}$
$C_{s}$	the set of clusters obtained from a load curve clustering on $\left[X_{0},X_{1},\ldots ,X_{s}\right]$
$C_{si}$	the *i*th cluster in $C_{s}$, $1\leq i\leq K_{s}$. The cluster center of $C_{si}$ is $\mu_{si}$
$n_{si}$	No. of daily load curves in $C_{si}$
$A_{s}$	the set of cluster centers, also called load patterns, of $C_{s}$
$\mu_{si}$	the *i*th load patterns in $A_{s}$, referring to the cluster center of $C_{si}$, $1\leq i\leq K_{s}$
$K_{s}$	No. of load patterns in $A_{s}$ / clusters in $C_{s}$
$P_{s}$	the set of probabilities of load patterns $A_{s}$
$p_{si}$	the probability of $\mu_{si}$, $p_{si}\in P_{s}$, $\sum_{i=1}^{K_{s}}p_{si}=1$
$X_{0}$	the set of initial daily load curves, $X_{0}\in X$
$X_{1}$	the first set of new daily load curves, $X_{1}\in X$
$A_{0}$	the set of load patterns obtained from a load curve clustering on $X_{0}$
$a_{1}$	the set of load patterns obtained from a load curve clustering on $X_{1}$
$iA_{1}$	the set of load patterns obtained from load pattern intergradtion on $\left[A_{0},a_{1}\right]$
$A_{1}$	the set of updated load patterns obtained from the incremental clustering on $\left[X_{0},X_{1}\right]$

**Table 3:** Summary of comparison methods.

Method	Description	Incremental	Probability Strategy (PS)
FCCWT	The method designed for daily load curve clustering [39]	no	no
*K*-means	The common *K*-means algorithm	no	no
**ICluster-PS**	The proposed method designed for daily load curve clustering	yes	yes
ICluster	The proposed method without PS	yes	no
I*K*-means-PS	The incremental method that adopts *K*-means with PS	yes	yes
I*K*-means	The incremental method that adopts *K*-means without PS	yes	no
HPStream	The algorithm for high-dimensional data streams [29]	yes	no

**Table 4:** Mean clustering performance comparison of the methods.

Method		t=3				t=6				t=9		
		DB${}^{-}$	DVI${}^{+}$	SWC${}^{+}$		DB${}^{-}$	DVI${}^{+}$	SWC${}^{+}$		DB${}^{-}$	DVI${}^{+}$	SWC${}^{+}$
FCCWT ${}^{*}$		0.9033	0.1267	0.5846		0.9066	0.1316	0.5854		0.8932	0.2056	0.5985
*K*-means ${}^{*}$		1.0333	0.1487	0.4778		1.0283	0.1519	0.4802		1.0058	0.2240	0.5110
**ICluster-PS**		**1.3159**	0.0624	**0.3350**		**1.2556**	0.0707	**0.3856**		**1.1599**	**0.1322**	**0.4199**
ICluster		1.3369	0.0596	0.3212		1.2821	0.0672	0.3457		1.1924	0.1317	0.3823
I*K*-means-PS		1.8056	0.0403	0.1129		1.6337	0.0576	0.2776		1.4048	0.0899	0.3655
I*K*-means		1.7872	0.0422	0.1193		1.6177	0.0578	0.2704		1.4390	0.0917	0.3435
HPStream		2.2739	**0.0740**	0.3314		1.9913	**0.0781**	0.3478		1.9131	0.0887	0.3506
**Improvement**		26.4%	−15.7%	1.1%		22.4%	−9.5%	10.9%		19.4%	44.2%	19.8%

${}^{*}$: non-incremental method; ${}^{-}$: the minimum is the optimal; ${}^{+}$: the maximum is the optimal.

**Table 5:** Mean error comparison of the methods.

Method		t=3				t=6				t=9		
		NRMSE	MAE	sMAPE		NRMSE	MAE	sMAPE		NRMSE	MAE	sMAPE
**ICluster-PS**		**0.0436**	**10.0744**	**0.0332**		**0.1008**	**20.3269**	**0.0784**		**0.1363**	**32.2105**	**0.1059**
ICluster		0.1172	28.7795	0.0907		0.1585	32.2034	0.1239		0.1820	40.9046	0.1424
I*K*-means-PS		0.0777	17.4945	0.0608		0.1520	29.9669	0.1210		0.1870	40.0440	0.1470
I*K*-means		0.1161	29.6179	0.0910		0.1939	38.5883	0.1533		0.2204	45.8908	0.1728
HPStream		0.2509	61.8134	0.1888		0.2489	58.7053	0.1942		0.2654	63.7557	0.2082
**Improvement**		62.4%	66.0%	63.5%		48.0%	47.3%	48.9%		38.2%	29.8%	38.7%

## Data Availability

The data used in this work are publicly available on OpenEI at https://openei.org/datasets/files/961/pub/.
